# Acute Hepatitis E: A Rare Cause of Acute Liver Failure in a Patient With Acute Myeloid Leukemia

**DOI:** 10.7759/cureus.10628

**Published:** 2020-09-24

**Authors:** Zachary Field, Michelle Russin, Rodrigo M Murillo Alvarez, Mario Madruga, Steve Carlan

**Affiliations:** 1 Internal Medicine, Orlando Regional Medical Center, Orlando, USA; 2 Pathology, Orlando Regional Medical Center, Orlando, USA; 3 Obstetrics and Gynecology, Orlando Regional Medical Center, Orlando, USA

**Keywords:** hepatitis e virus, liver function, acute myeloid leukemia (aml)

## Abstract

Immunocompromised patients are particularly at risk to develop hepatitis E virus (HEV) infection and its related complications. We present a rare case of HEV infection in a 35-year-old Hispanic female with concomitant acute myeloid leukemia (AML). The patient presented with acute liver failure within a few weeks after receiving a blood transfusion.

Our case likely represented an acute de novo HEV infection after chemotherapy in a patient with concurrent AML, evidenced by the presence of anti-HEV IgM antibodies as well as histological findings, and with a previous history of recent transfusions being one of the strongest risk factors for transmission. Liver failure from an acute de novo hepatitis E infection with concurrent AML can be catastrophic in the immunosuppressed patient. Our case is particularly unique due to the uncommon presentation of acute hepatitis E in a non-pregnant reproductive aged Hispanic female with recently diagnosed AML. Clinicians should maintain a low threshold to test serum HEV-RNA if a patient presents with signs and symptoms suggestive of acute hepatitis.

## Introduction

Hepatitis E viral (HEV) infection has a worldwide distribution and usually has a self-limited clinical course [1-3]. In most patients, an estimated recovery occurs within four to six weeks, but immunocompromised patients are at risk of developing symptomatic acute hepatitis or even chronic infection [4,5]. Fecal contamination is the most common mechanism of transmission in developing countries due to poor sanitary conditions [1], while in developed countries animal reservoir transmission and transfusion-related transmission have been reported [6].

## Case presentation

A 35-year-old Hispanic female with a past medical history significant only for acute myeloid leukemia (AML), who had previously undergone chemotherapy and consolidation therapy, presented for evaluation of progressively worsening jaundice over the past seven days. She also reported clay-colored stools that developed the day prior to admission, as well as generalized symptoms of nausea, chills, and malaise. Regarding her AML, the patient was diagnosed five months prior to admission with cytogenetics revealing a t(8;21) translocation. She underwent induction chemotherapy with 7 + 3 regimen. She then had three cycles of consolidation therapy with high-dose cytosine arabinoside. The patient was originally from Mexico, but had been living in the United States for 16 years.

The physical exam was only significant for scleral icterus and jaundice. Laboratory values are seen in Table I and were notable for an aspartate aminotransferase (AST) of 3,846 U/L (13-39 U/L), alanine aminotransferase (ALT) of 3,346 U/L (7-52 U/L), alkaline phosphatase of 189 U/L (34-104 U/L), and total bilirubin of 13.3 mg/dL [0.3-1.0 mg/dL]. An acute hepatitis panel was ordered, which tested for hepatitis A, hepatitis B, and hepatitis C antibodies as well as hepatitis B surface antigen, and was negative.

**Table 1:** Laboratory values

Basic Lab Results and Anticoagulation Studies						
Test		Result	Flag	Units	Reference Interval	
Complete metabolic panel	Sodium, serum	134	High	mmol/L	136-145	
	Potassium, serum	3.5	Low	mmol/L	3.6-5.1	
	Chloride, serum	103	-	mmol/L	98-107	
	CO_2_ content	24	-	mmol/L	22-32	
	Blood urea nitrogen (BUN)	5	Low	mg/dL	8-20	
	Creatinine, serum	0.5	-	mg/dL	0.5-1.0	
	BUN/creatinine ratio	10	-	Ratio	7.3-21.7	
	Glucose serum	133	High	mg/dL	65-100	
	Anion gap	7	-	mmol/L	2-12	
	Calcium, serum	8.5	Low	mg/dL	8.6-10.3	
	Aspartate aminotransferase	3,846	High	IU/L	13-39	
	Alanine aminotransferase	3,346	High	IU/L	7-52	
	Alkaline phosphatase	189	High	IU/L	34-104	
	Bilirubin, total	13.3	High	mg/dL	0.3-1.0	
	Protein total, serum	6.1	Low	g/dL	6.5-8.9	
	Albumin, serum	2.8	Low	g/dL	3.5-5.7	
	Osmolality, calculated	280	-	mOs/kg	280-300	
	Bilirubin, direct	8.1	High	mg/dL	0.0-0.2	
Complete blood count	White blood cell count	4.8	-	×10^3^/μL	4.1-10.4	
	Red blood cell count	3.71	Low	×10^6^/μL	3.9-5.0	
	Hemoglobin	12.6	-	g/dL	11.8-15.1	
	Hematocrit	36.8	-	%	34.0-44.0	
	Platelet count	65	Low	×10^3^/μL	145-355	

**Figure 1:**
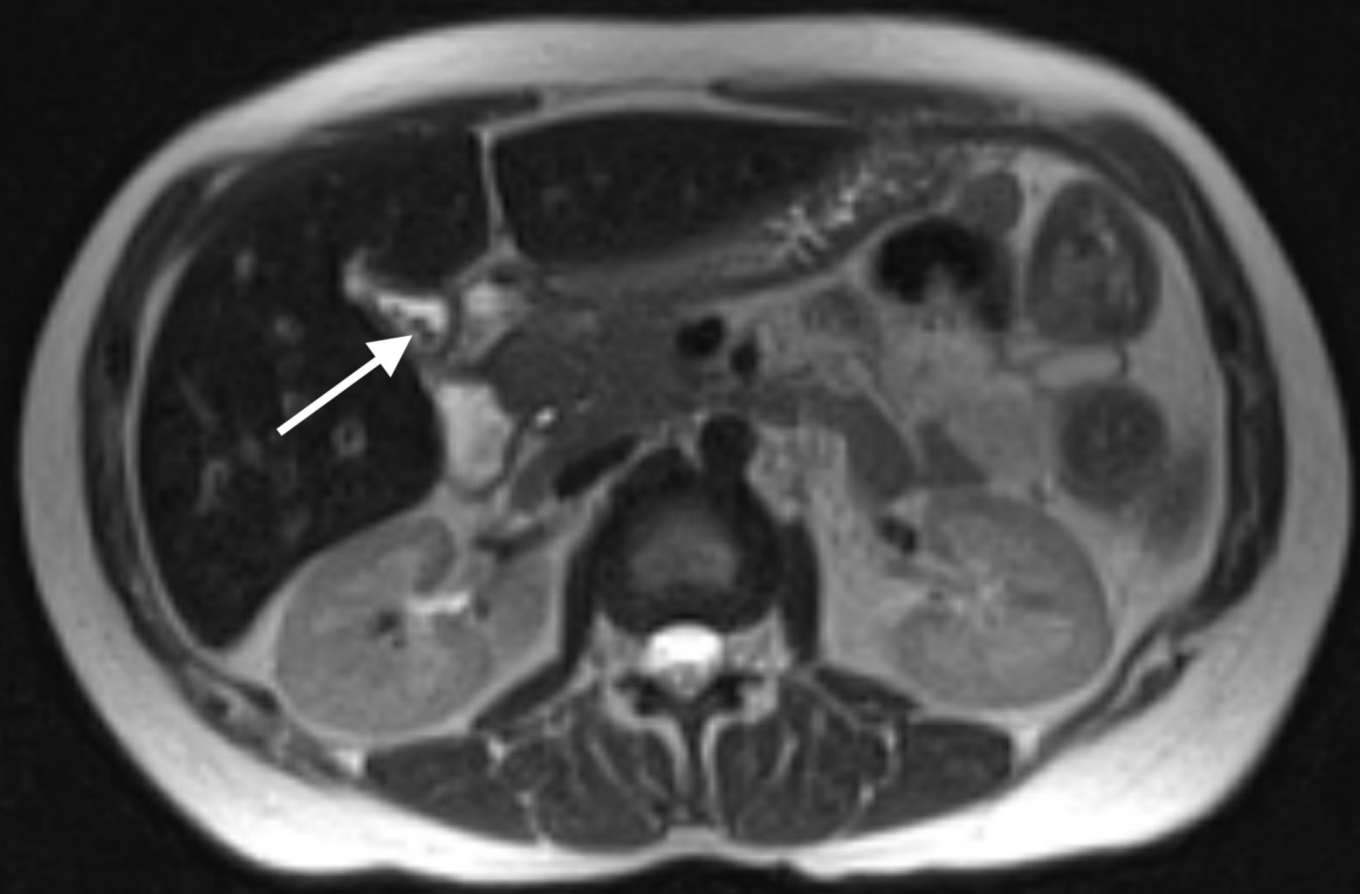
Magnetic resonance cholangiopancreatography (MRCP) Stones (marked with arrow) and sludge within a contracted gallbladder. Gallbladder wall measuring 3 mm, the upper limit of normal. Diffuse signal changes of the liver consistent with iron overload. No focal liver lesions are seen.

Her liver function tests began to improve, but the etiology of her acute liver failure remained unclear. The patient underwent magnetic resonance cholangiopancreatography (MRCP), which showed some sludge and gallstones but no pericholecystic fluid or evidence of an impacted stone (Figure 1).

Further workup including antinuclear antibody (ANA), anti-mitochondrial antibody, anti-smooth muscle antibody, HIV antigen, acetaminophen level, ceruloplasmin level, as well as cytomegalovirus polymerase chain reaction (PCR), Epstein-Barr antibodies, herpes-simplex PCR, and hemochromatosis gene (HFE) gene analysis were all negative or within normal limits.

**Figure 2:**
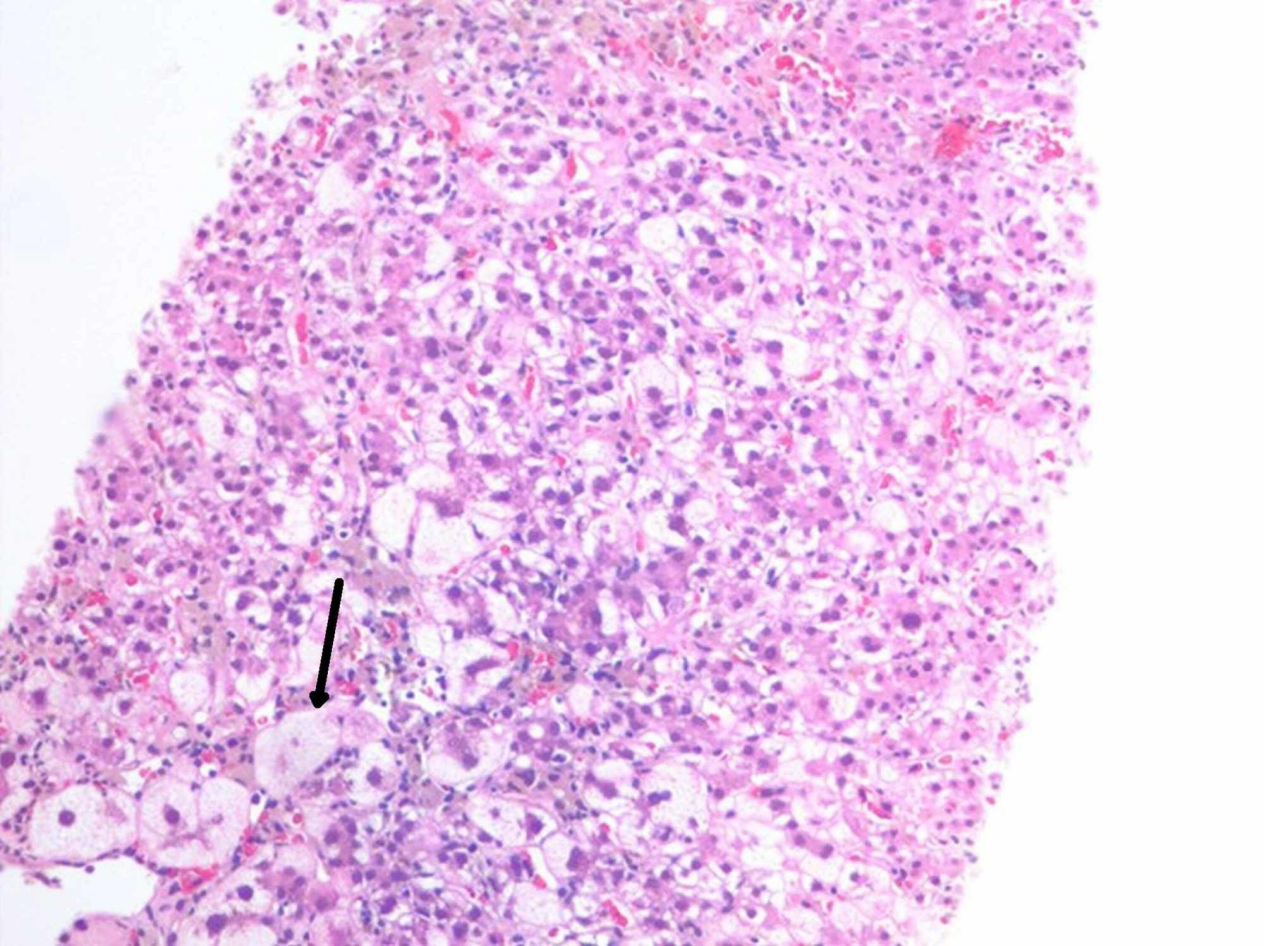
Core needle biopsy of the liver Extensive ballooning degeneration (arrow) with lobular disarray and individual hepatocellular necrosis.

Iron studies were ordered that revealed a ferritin of 88 ng/mL (10.0-291.0 ng/mL), serum iron of 240 µg/dL (28-170 µg/dL), total iron-binding capacity of 209 µg/dL (255-450 µg/dL), transferrin saturation of 115% (20%-50%), and transferrin of 149 mg/dL (192-382 mg/dL). The hematology and oncology service was consulted and did not believe that her presentation was related to chemotherapy toxicity. Gastroenterology was consulted who recommended that the patient undergo a liver biopsy, which showed extensive ballooning degeneration with lobular disarray and evidence of necrosis (Figure 2).

Additional stains including trichrome and reticulin stains were used to rule out increased fibrosis. 

On the seventh day of hospitalization, laboratory results for HEV immunoglobulin M (IgM) antibodies were reported positive. Upon further questioning, the patient stated that she was from León, Guanajuato, located in central Mexico. She denied any prior exposure to swine or other farm animals, drinking from contaminated water sources, or consuming liver. She did state that she had received approximately 10-15 units of packed red blood cells over the past five months prior to her admission for severe anemia. Her low hemoglobin was the initial laboratory abnormality requiring workup that lead to her diagnosis of AML. Her most recent blood transfusion occurred 16 days prior to her development of jaundice. 

When her symptoms and laboratory values continued improving, she was discharged from the hospital with close follow-up by hematology, oncology, and gastroenterology. She had serial comprehensive metabolic panels over the following weeks to monitor her liver function. Her ALT and AST eventually returned completely to her baseline three weeks after discharge.

## Discussion

HEV is a major cause of viral hepatitis worldwide and is especially endemic in many parts of the developing world. However, hepatitis E is becoming increasingly recognized in the developed world, particularly through zoonotic transmission of genotype 3 in the United States [7]. Groups typically at high risk in developed countries are males over the age of 50 years [7,8]. Acute infection typically causes a self-limiting hepatitis that may progress to fulminant hepatitis in pregnant women [8]. More recently, rare cases of acute hepatitis with progression to chronic infection and cirrhosis have been demonstrated in immunocompromised patients [9]. Cases in patients with solid organ transplants, HIV infections, and with hematologic malignancies have been reported [10]. In industrialized countries, these infections are almost exclusively caused by genotype 3 in the immunosuppressed population [7]. Therefore, acute hepatitis E infection should be considered in patients presenting with symptomatic liver failure or abnormal liver function tests, especially with a background of malignancy, even without travel to endemic regions.

Our case is particularly unique due to the uncommon presentation of acute hepatitis E in a non-pregnant reproductive aged Hispanic female with recently diagnosed AML. This likely represents an acute de novo infection evidenced by the presence of IgM anti-HEV as well as histological findings of focal necrosis, ballooned hepatocytes, and acidophilic degeneration of hepatocytes. Approximately 90% of patients will test positive for IgM anti-HEV at two weeks after infection and will remain positive for up to five months later, indicating our patient was recently infected [7]. Recent studies have suggested that active hepatitis E infections in patients on immunosuppressive therapy regimens, including stem cell transplants or chemotherapy, are typically acquired de novo and do not represent reactivation of latent hepatitis E [10,11]. There are few reports documenting the burden of hepatitis E in patients with hematologic malignancies but noted it may be associated with serious complications in the immunosuppressed population such as liver failure and mortality [12]. One case-controlled study found that the seroprevalence of immunoglobulin G (IgG) and IgM antibodies to HEV were found to be significantly higher in cancer patients than in controls (26.0% vs. 13.0%; p < 0.001). However, no studies to date have systematically assessed the exact prevalence or incidence of HEV co-infection among hematological patients [1]. Among the limited number of literature reports in cancer patients, it has usually been documented as chronic HEV infections, as opposed to the acute diagnosis in our patient case. Of these cases previously reported in the literature, they include a patient with untreated hairy cell leukemia, a patient with idiopathic cluster of differentiation 4 (CD4) thymus cell (T cell) lymphopenia, and patients treated for lymphoma, chronic myelomonocytic leukemia, and bursa of Fabricius cell (B cell) chronic lymphocytic leukemia [8]. To the best of our knowledge, however, there have been no reported cases of patients with AML who had previously undergone chemotherapy presenting with an acute hepatitis E infection in the United States, such as our patient.

Due to the recently discovered rising number of hepatitis E cases in immunocompromised patients, clinicians should maintain a low threshold to test serum HEV-RNA if a patient presents with signs and symptoms suggestive of acute hepatitis. Cancer patients are a high-risk population who should be informed to avoid exposure to sources of HEV infection, such as raw or undercooked foods and animal exposure [1]. In the event of HEV infection, reduction of immunosuppression has been reported successful as treatment but if not feasible or unsuccessful, ribavirin should be prescribed [8,9,12]. While clearance is usually spontaneous in immune-competent individuals, these at-risk groups may develop a more complicated and protracted disease course. The hepatic damage produced by the HEV can result in the production and regeneration of growth factors and may evolve into cirrhosis, putting a patient at further risk for hepatocellular carcinoma, especially in an immunosuppressed patient [1]. 

The incubation period for HEV is two to six weeks [8]. Given that our patient’s symptoms developed 16 days after her last transfusion and she lacked other risk factors for contracting HEV, it is likely that she acquired the infection through a contaminated blood product transfusion. Blood product donors are not currently screened for HEV, yet there are increasing numbers of reported transmissions of acute and chronic infections in blood transfusion recipients. While knowing immunosuppressed patients are already at higher risk for acquiring viral infections, clinicians should be aware that the administration of transfused blood products may further increase the risk of HEV. Infection with HEV during the immunocompromised state can significantly alter the patient’s disease course [12]. Close follow-up with any immunocompromised patient for either spontaneous viral clearance or resolution of symptoms to determine the need for therapeutic intervention is therefore recommended.

## Conclusions

Although the majority of HEV infections occur in developing countries, cases in the developed world do occur and several cases have been well documented, particularly in immunocompromised hosts unable to effectively clear the infection. In this group, a high index of suspicion of HEV infection is important, especially when clinical manifestations of acute hepatitis, abnormal liver function studies, or a medical history of a hematological malignancy is present. The diagnosis is usually achieved by the identification of HEV antibodies or HEV-RNA; however, a liver biopsy can be justified in order to assess severity and evolution of the disease and to rule out other causes. A close follow-up is required to determine the need for additional interventions until the infection is entirely cleared.
